# Long-term neurotoxicity among childhood acute lymphoblastic leukaemia survivors enrolled between 1971 and 1998 in EORTC Children Leukemia Group studies

**DOI:** 10.1007/s12672-024-00869-6

**Published:** 2024-01-29

**Authors:** Maëlle de Ville de Goyet, Michal Kicinski, Stefan Suciu, Els Vandecruys, Anne Uyttebroeck, Alina Ferster, Claire Freycon, Geneviève Plat, Caroline Thomas, Mélissa Barbati, Marie-Françoise Dresse, Catherine Paillard, Claire Pluchart, Pauline Simon, Christophe Chantrain, Odile Minckes, Jutte van der Werff ten Bosch, Yves Bertrand, Pierre Rohrlich, Frederic Millot, Robert Paulus, Yves Benoit, Caroline Piette, the European Organisation for Research, Treatment of Cancer (EORTC) Children’s Leukemia Group (CLG)

**Affiliations:** 1 Department of Paediatric Haematology-Oncology, Cliniques Universitaires Saint-Luc, Brussels, Belgium; 2 EORTC Headquarters, Brussels, Belgium; 3 Department of Paediatric Haematology-Oncology, Ghent University Hospital, Ghent, Belgium; 4 Department of Paediatric Haematology-Oncology, University Hospital Leuven, Louvain, Belgium; 5 Department of Haemato Oncology, HUDERF (ULB), Brussels, Belgium; 6 Department of Paediatric Haematology-Oncology, CHU Grenoble, Grenoble, France; 7 Department of Haematology, CHU Toulouse, Toulouse, France; 8 Department of Haematology, CHU Nantes, Nantes, France; 9 Department of Paediatric Haematology-Oncology, CHRU Lille, Lille, France; 10 Department of Paediatrics, University Hospital Liège and University of Liège, Liège, Belgium; 11 Department of Paediatric Haematology-Oncology, CHRU Strasbourg, Strasbourg, France; 12 Department of Paediatric Haematology and Oncology, CHU Reims, Reims, France; 13 Pediatric Oncology, CHRU Besançon, Besançon, France; 14 Division of Paediatric Haematology-Oncology, CHC MontLégia, Liège, Belgium; 15 Department of Paediatric Haematology-Oncology, CHU Caen, Caen, France; 16 VUB, Brussels, Belgium; 17 Department of Paediatric Onco-Haematology, Lyon University Hospital, Hospices Civils de Lyon and, Université Claude Bernard Lyon, Lyon, France; 18 Division of Paediatric Haematology-Oncology, CHU Nice, Nice, France; 19 Department of Paediatric Haematology-Oncology, CHU Poitiers, Poitiers, France; 20 CHR Verviers East Belgium, Verviers, Belgium; 21 Service de Pédiatrie, CHU Liège, Avenue de l’Hôpital 1, 4000 Liège, Belgium

## Abstract

Survival after childhood acute lymphoblastic leukemia (ALL) has increased over the last 40 years with an overall survival above 90%. Survivors may experience neurological late effects secondary to chemotherapy and radiotherapy. This observational retrospective study evaluated the cumulative incidence of neurological late effects among 890 childhood ALL survivors treated in EORTC CLG trials (58741, 58831/2 and 58881) between 1971 and 1998. Median follow-up was 19 years and interquartile range of the follow-up was 15–22 years. At 20 years from the end of treatment, approximately 66% of patients from the 58741 trial (accrual time: 1971–1978) and approximately 15% from the more recent trials had cognitive disturbance grade 1 or higher. Cumulative incidences at 20 years from treatment end of seizures, stroke and leukoencephalopathy were respectively 45%, 16% and 62% in study 58741, 13%, 2% and 5% in study 58831/2, and 8%, 2% and 3% in study 58881. Patients who were 10–17 years of age at diagnosis had a higher incidence of stroke and leukoencephalopathy as compared to those less than 6 years of age. Noteworthy, all neurological late effects continued to occur beyond 5 years after end of treatment. This retrospective study highlights the frequency of neurological late effects in survivors of childhood ALL. With the increase of the overall survival of ALL patients, the role and potential benefit of longitudinal neurological screening should be evaluated in further studies as these neurological late effects become an important public health challenge. This study is part of the larger EORTC CLG 58 Late Adverse Effects (LAE) study (ClinicalTrials.gov Identifier NCT01298388, date of registration February 16, 2011).

**Supplementary Information:**

The online version contains supplementary material available at 10.1007/s12672-024-00869-6.

## Introduction

Survival after childhood acute lymphoblastic leukaemia (ALL) has increased over the last 40 years [1]. The expected cure rate is currently greater than 90%, and the attention is now focused on the long-term outcome of the survivors. Indeed, a proportion of survivors experience long-term treatment-related complications in health, behaviour and/or quality of life, and the majority receive general medical care, with infrequent coverage of cancer treatments-related late effects screening [2]. 

Various neurocognitive outcomes are reported in survivors of childhood acute lymphoblastic leukaemia (SCALL) including neurocognitive disturbance but also symptomatic or not leukoencephalopathy, seizure or stroke.

Impairment rates across neurocognitive domains at mean 26 years since diagnosis ranged from 28.6% to 58.9% in a large cohort of SCALL [3]. Reported risk factors for cognitive disturbance are cranial radiotherapy (CRT), prednisone, dexamethasone, high dose intravenous Methotrexate (HD MTX) and intrathecal Methotrexate (IT MTX) [3–7]. Cognitive disturbances are related to functional outcomes in adulthood, including college graduation, full-time employment rates and health-related quality of life [3, 4, 8]. 

Seizure and stroke are also reported late effects in SCALL. The 10-year cumulative incidence of subsequent seizure and/or stroke in SCALL was estimate at 4.3% [9]. Seizures can be caused by MTX but also asparaginase [10]. Asparaginase is also well-known for causing acute neurotoxicity such as intracranial thrombosis [11]. The long-term neurological consequence of this acute toxicity remains to be clarified and is difficult to evaluate separately from other neurotoxic drugs. Acute neurotoxicity of methotrexate is also well-known and may cause transient stroke-like symptoms, encephalopathy, seizure and/or aphasia but can also cause long-term leukoencephalopathy. Higher cumulative number of intrathecal therapies (ITTs) could also be a risk factor for leukoencephalopathy [12]. Finally, cytarabine neurotoxicity such as leukoencephalopathy is also reported and high dose intravenous (IV) cytarabine may cause seizure, cerebral dysfunction or acute cerebellar syndrome [13]. 

The goal of our follow-up study is an overall assessment of late neurotoxicity in SCALL treated in three consecutive clinical trials from the European Organisation for Research and Treatment of Cancer (EORTC) Children Leukemia Group (CLG) between 1971 and 1998. For this purpose, we studied the incidence of four neurological late adverse effects (cognitive disturbance, seizures, stroke and leukoencephalopathy) and their trends over the three consecutive trials. We also investigated associations between patients’ and treatment characteristics and these neurological late adverse effects. This study is part of the larger EORTC CLG 58 Late Adverse Effects (LAE) study (ClinicalTrials.gov Identifier NCT01298388), assessing the long-term outcomes of childhood ALL survivors [14].

## Methods

### Patient population and data collection

The study participants included childhood ALL patients (less than 18 years at diagnosis) who were treated in one of the EORTC CLG clinical trials 58741 (accrual period: 1971–1978), 58831/2 (1983–1989), and 58881 (1989–1998) and were alive two months after the end of the first line therapy (Figure S1). Exclusion criteria in these initial studies were B-cell non-Hodgkin lymphomas, B-cell ALL, (except in 58741 study), previously treated patients (corticoids and/or vincristine for less than 8 days were allowed in 58831/2 studies, corticoids for less than 8 days were allowed in 58881 study), severe encephalopathy, severe heart disease or trisomy 21 (in 58831/2 studies), initial CNS involvement (in 58832 study) and criteria related to risk-group definitions (in 58831/2 studies). Patients who were not eligible for participation in these trials were excluded from the present neurotoxicity study, except in case the initial exclusion criteria was a central nervous system (CNS) involvement at baseline. Patients with a history of trisomy 21 were excluded as well, irrespective of whether it was an exclusion criterion.

Between 2011 and 2018, 24 hospitals from France and Belgium participated in the 58LAE study and provided updated follow-up information about eligible patients by filling in an electronic case report form (eCRF), based on medical records of the patients. New clinical evaluation was recommended but not mandatory. The eCRF comprised information about clinical outcomes, treatments, and long-term toxicities, including neurotoxicity (cognitive disturbance, seizures, strokes and leukoencephalopathy).

### Treatment protocols

In this section, we provide details on definitions (i.e., CNS involvement), treatments (i.e., CRT, hematopoietic stem cell transplantation [HSCT] and high-dose cytarabine [HD Cytarabine]) and risk group (very high-risk [VHR] in study 58881) considered for the study of association with neurotoxicity (Table 1). Other details regarding studies 58741, 58831/2 and 58881 are reported in Supplementary Table S1) [15].

**Table 1:** Descriptive statistics by protocol

	Protocol			Total (N = 890)
	58741 (N = 60)	58831/2 (N = 199)	58881 (N = 631)	
	N (%)	N (%)	N (%)	N (%)
**Sex**				
Male	30 (50.0)	104 (52.3)	337 (53.4)	471 (52.9)
Female	30 (50.0)	95 (47.7)	294 (46.6)	419 (47.1)
**Age at diagnosis, years**				
< 6	37 (61.7)	125 (62.8)	418 (66.2)	580 (65.2)
6–9	14 (23.3)	47 (23.6)	120 (19.0)	181 (20.3)
10–17	9 (15.0)	27 (13.6)	93 (14.7)	129 (14.5)
**WBC at diagnosis, × 10**^**9**^**/l**				
< 25	41 (68.3)	139 (69.8)	432 (68.5)	612 (68.8)
25 ≤50	9 (15.0)	30 (15.1)	77 (12.2)	116 (13.0)
≥ 50	10 (16.7)	30 (15.1)	122 (19.3)	162 (18.2)
**CNS involvement at diagnosis**				
N available data	57 (95.0)	197 (99.0)	628 (99.5)	882 (99.1)
No CNS involvement	55 (96.5)	192 (97.5)	588 (93.6)	835 (94.7)
CNS involvement	2 (3.5)	5 (2.5)	40 (6.4)	47 (5.3)
**NCI risk group**				
Standard Risk	42 (70.0)	145 (72.9)	431 (68.3)	618 (69.4)
High Risk	18 (30.0)	54 (27.1)	200 (31.7)	272 (30.6)
**CRT**				
No	0 (0.0)	153 (76.9)	591 (93.7)	744 (83.6)
Yes	60 (100.0)	46 (23.1)	40 (6.3)	146 (16.4)
**HSCT**				
No	60 (100.0)	182 (91.5)	549 (87.0)	791 (88.9)
Yes	0 (0.0)	17 (8.5)	82 (13.0)	99 (11.1)
**HSCT conditioning**				
N available data	0 (0.0)	16 (8.0)	80 (12.7)	96 (10.8)
HDCT only	0 (0.0)	2 (12.5)	11 (13.8)	13 (13.5)
TBI + HDCT	0 (0.0)	14 (87.5)	69 (86.3)	83 (86.5)
**HSCT in first line treatment**				
No	60 (100.0)	195 (98.0)	615 (97.5)	870 (97.8)
Yes	0 (0.0)	4 (2.0)	16 (2.5)	20 (2.2)
**VHR after consolidation in protocol 58881**				
N available data	–	–	614 (97.3)	
No	–	–	564 (91.9)	–
Yes	–	–	50 (8.1)	–
**HD-Cytarabine arm in protocol 58881**				
N randomized patients	–	–	207 (32.8)	–
No HD Cytarabine	–	–	109 (52.7)	–
HD Cytarabine	–	–	98 (47.3)	–
**Relapse**				
No	36 (60.0)	166 (83.4)	514 (81.5)	716 (80.4)
Yes	24 (40.0)	33 (16.6)	117 (18.5)	174 (19.6)
**CNS relapse**				
No	42 (70.0)	184 (92.5)	579 (91.8)	805 (90.4)
Yes	18 (30.0)	15 (7.5)	52 (8.2)	85 (9.6)
**Survival status**				
Alive	42 (70.0)	183 (92.0)	579 (91.8)	804 (90.3)
Dead	18 (30.0)	16 (8.0)	52 (8.2)	86 (9.7)

CNS, central nervous system; CRT, cranial radiotherapy; HD Cytarabine, high-dose cytarabine; HSCT, hematopoietic stem cell transplantation; NCI, National Cancer Institute; TBI, total body irradiation; VHR, very high risk; WBC, white blood cells.

CNS involvement was defined as the presence of ≥ 1 blast on cytospin with any white blood cells count (WBC) in the cerebrospinal fluid. CRT was administered to all patients in study 58741. Then, studies 58831/2 investigated whether CRT could be omitted with systemic and intrathecal CNS prophylaxis in CNS-negative patients. Based on these results, CRT was fully omitted in the 58881 study. In 58881 study, patients with VHR criteria (≥ 1000 blasts/mm^3^ of blood at the end of prephase, and/or who did not achieve complete remission after induction and/or with t(4;11)/MLL-AF4) received an intensified treatment according to the BFM relapse protocol. HSCT was reserved to VHR patients enrolled in study 58881 in first remission if a donor was available. Finally, the value of HD Cytarabine (1 g/m^2^/day intravenously on days 8–9, 22–23, 36–37 and 50–51 of interval therapy) was evaluated in a randomized way in increased risk patients in complete remission in 58881 study.

### Ethics

At the time of the enrolment in studies 58741, 58831/2 and 58881, informed consent was sought from all participants or, if participants were under 18, from a parent and/or legal guardian, according to local practice of each participating centre and in accordance with the Declaration of Helsinki. The EORTC study 58LAE was approved by the Ethical Committees of the participating institutions and informed consent was obtained from all alive patients at the time of the latest follow-up, in accordance with the applicable national legislation.

### Definitions and criteria of evaluation

Late adverse effects were defined as toxicities occurring during first line therapy and still present two months after the end of first line therapy, or appearing two months or later after the end of first line therapy. Definitions of the adverse effects (and grades for cognitive disturbance) were based on the Common Terminology Criteria for Adverse Events (CTCAE), Version 4.0 [16]. 

Time to events of interest (leukoencephalopathy, seizure and stroke) was defined as the time between two months after the end of the first line therapy and a diagnosis of the corresponding event.

### Statistical analysis

For all estimated parameters, point estimates and 2-sided 95% confidence intervals (CI) are presented. All tests were performed at a two-sided significance level of 0.05.

The cumulative incidence of leukoencephalopathy, seizures and stroke was estimated using the Aalen-Johansen estimator [17]. The proportional sub-distribution hazards model was used to investigate the association between protocol, age at diagnosis, and CNS involvement at diagnosis, and the cumulative incidence of the neurotoxicities [18]. Patients with an event of interest before two months after the end of the first line therapy were counted as events at day 1. Death without the event of interest was considered as a competing event. The follow-up of patients was censored at the time of last visit. In case of a missing event time in the analysis of leukoencephalopathy, seizures and stroke, the cumulative incidence functions were estimated using multiple imputation. In the analyses of cumulative incidences, time to event was imputed using the estimated cumulative incidence function for the group of interest based on complete cases. The estimates based on the imputed data were then combined using the Rubin’s rules [19]. Multiple imputation with the asymptotic normal data augmentation scheme was used to obtain estimates from the Fine and Gray model [20]. The number of imputations was 100 in the estimation of the cumulative incidence functions and 10 in the estimation of the subdistribution hazard ratio. Logistic regression was used to investigate the association between age at diagnosis, CNS involvement at diagnosis, HSCT, HD Cytarabine dose (among patients from the 58881 study only), and VHR (among patients from the 58881 study only) and cognitive disturbance. Both in the logistic regression and the Fine and Gray model analyses, for each covariate of interest a separate model was fitted. The analyses of age, CNS involvement at diagnosis, and HSCT were adjusted by protocol. Patients with missing covariates that were required for a specific analysis were excluded from such analysis.

A Fine and Gray model stratified by protocol with a time-varying covariate for HSCT taking a value 1 after HSCT and a value 0 otherwise was used to investigate the association between HSCT and seizures. Since the relapse status and HSCT were correlated, a sensitivity analysis was performed in which relapse modelled as a time-varying covariate taking a value 1 after relapse and a value 0 otherwise was added to this model.

## Results

### Patients and treatment characteristics

Among 3228 patients included in the EORTC 58741, 58831/2, and 58881 trials, 2329 patients were eligible for participation in the current study (Figure S1). Among those eligible, data for at least one neurotoxicity outcome was available for 890 patients, who were included in the analysis. Patients with and without available data were similar in terms of the National Cancer Institute (NCI) risk group and exposure to CRT and HSCT (Table S2). However, death and relapse were reported more often for patients without than those with available data.

Among the 890 patients included in this study, the median follow-up was 19 years and the interquartile range of the follow-up was 15–22 years. Relapse occurred in 20% of the included patients, CNS relapse in 10%, 16% received CRT and 11% underwent HSCT (Table 1).

### Cognitive disturbance

Data on cognitive disturbance was available for 584 patients. Approximately 66% of patients from the 58741 trial and approximately 15% from the more recent trials had cognitive disturbance grade 1 or higher (Table 2). Adjusting for protocol, the associations of age at diagnosis, CNS involvement at diagnosis, and HSCT with the risk of cognitive disturbance were not statistically significant (Table 3). There was also no evidence of an association between VHR after consolidation in the 58881 study or being randomized to the HD Cytarabine arm in the 58881 study and the risk of cognitive disturbance.

**Table 2:** Neurotoxicity outcomes by protocol

	Protocol			Total (N = 890)
	58741 (N = 60)	58831/2 (N = 199)	58881 (N = 631)	
	N (%)	N (%)	N (%)	N (%)
**Cognitive disturbance**				
N available data	29 (48.3)	111 (55.8)	444 (70.4)	584 (65.6)
No	10 (34.5)	93 (83.8)	376 (84.7)	479 (82.0)
Yes, all CTCAE grades	19 (65.5)	18 (16.2)	68 (15.3)	105 (18.0)
Yes, CTCAE grade 1	6 (20.7)	9 (8.1)	47 (10.6)	62 (10.6)
Yes, CTCAE grade 2	7 (24.1)	5 (4.5)	20 (4.5)	32 (5.5)
Yes, CTCAE grade 3	6 (20.7)	4 (3.6)	1 (0.2)	11 (1.9)
**Seizures status**				
N available data	50 (83.3)	189 (95.0)	596 (94.5)	835 (93.8)
No event	24 (48.0)	156 (82.5)	520 (87.2)	700 (83.8)
Seizures	23 (46.0)	27 (14.3)	45 (7.6)	95 (11.4)
Death without seizures	3 (6.0)	6 (3.2)	31 (5.2)	40 (4.8)
**Stroke status**				
N available data	36 (60.0)	172 (86.4)	583 (92.4)	791 (88.9)
No event	26 (72.2)	161 (93.6)	537 (92.1)	724 (91.5)
Stroke	7 (19.4)	4 (2.3)	12 (2.1)	23 (2.9)
Death without stroke	3 (8.3)	7 (4.1)	34 (5.8)	44 (5.6)
**Leukoencephalopathy status**				
N available data	18 (30.0)	129 (64.8)	479 (75.9)	626 (70.3)
No event	4 (22.2)	115 (89.1)	438 (91.4)	557 (89.0)
Leukoencephalopathy	12 (66.7)	8 (6.2)	16 (3.3)	36 (5.8)
Death without leukoencephalopathy	2 (11.1)	6 (4.7)	25 (5.2)	33 (5.3)

CTCAE, Common Toxicity Criteria for Adverse Events.

**Table 3:** Associations between patients and treatment characteristics and cognitive disturbance

Covariate	Cognitive disturbance N (%)	No cognitive disturbance N (%)	Odds ratio (95% CI)	p-value
**Protocol**				0.001
58741	19 (65.5)	10 (34.5)	1	
58831/2	18 (16.2)	93 (83.8)	0.10 (0.04–0.26)	
58881	68 (15.3)	376 (84.7)	0.09 (0.04–0.21)	
**Age at diagnosis, years**				0.510*
< 6	75 (18.8)	323 (81.2)	1	
6–9	19 (17.9)	87 (82.1)	0.90 (0.50–1.62)*	
10–17	11 (13.8)	69 (86.3)	0.66 (0.32–1.34)*	
**CNS involvement at diagnosis**				0.536*
No CNS involvement	98 (17.8)	452 (82.2)	1	
CNS involvement	6 (21.4)	22 (78.6)	1.36 (0.52–3.55)*	
**HSCT**				0.233*
No	94 (17.7)	438 (82.3)	1	
Yes	11 (21.2)	41 (78.8)	1.54 (0.76–3.14)*	
VHR after consolidation**				0.682
Not VHR	63 (15.7)	339 (84.3)	1	
VHR	4 (12.9)	27 (87.1)	0.80 (0.27–2.36)	
Randomized HD Cytarabine**				0.861
No HD Cytarabine	15 (20.0)	60 (80.0)	1	
HD Cytarabine	13 (18.8)	56 (81.2)	0.93 (0.41–2.12)	

CNS, central nervous system; HD Cytarabine, high-dose cytarabine; HSCT, hematopoietic stem cell transplantation; VHR, very high risk.

*Adjusted for protocol

**Based on the 58881 study only

### Seizures

**Fig. 1:**
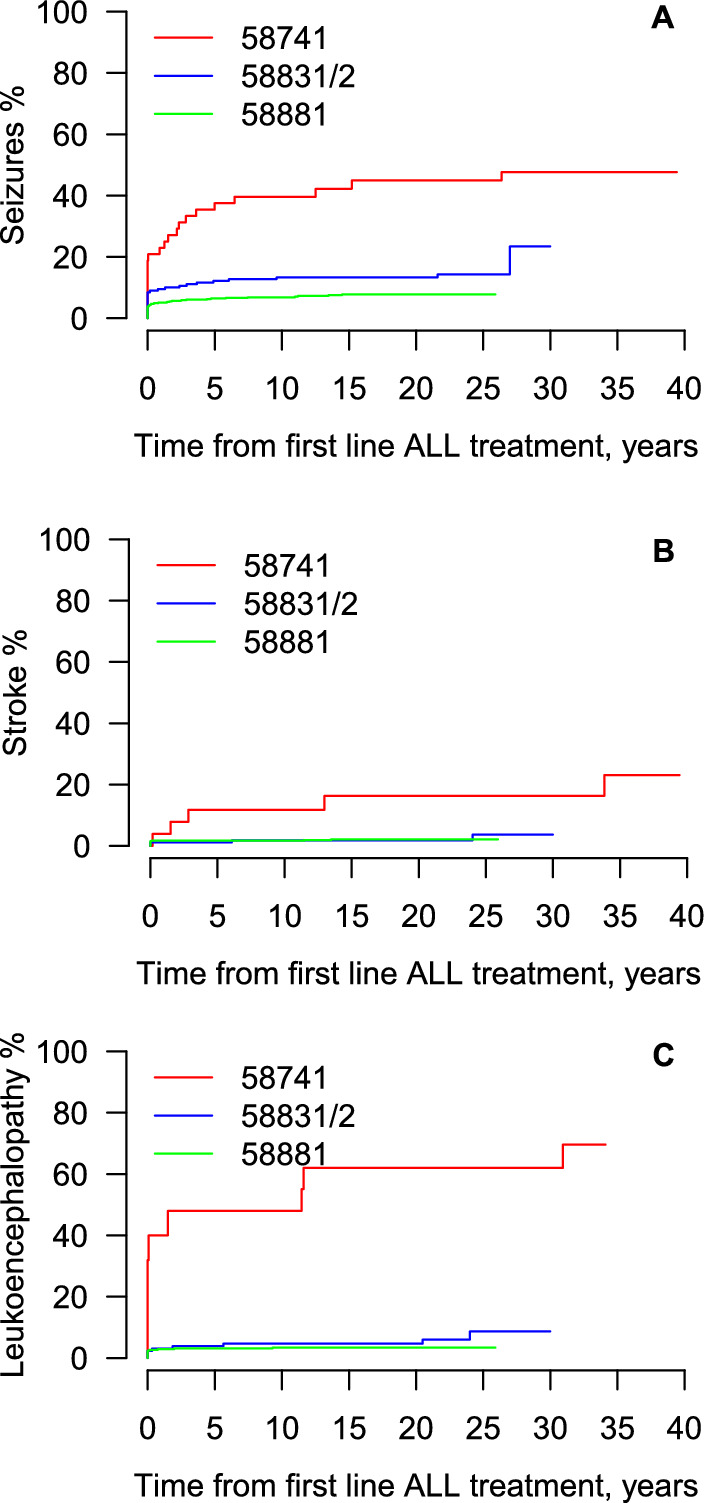
Cumulative incidence of seizures (**A**), stroke (**B**), and leukoencephalopathy (**C**) by protocol

The cumulative incidence of seizures at 20 years from end of treatment was 45% (95% CI 33–62%) in the 58741 study, 13% (95% CI 9–19%) in the 58831/2 studies and 8% (95% CI 6–10%) in the 58881 study (Fig. 1A). In a model adjusting for protocol, HSCT was associated with a higher incidence of seizures (HR = 3.09, 95% CI 1.50–6.35). In a model including protocol, relapse, and HSCT, relapse was strongly associated with a higher incidence of seizures (HR = 4.12, 95% CI 2.27–7.48) but the association for HSCT became weaker and not statistically significant (HR = 1.47, 95% CI 0.64–3.37). Table 4 presents the incidence of seizures by age at diagnosis, CNS involvement at diagnosis, VHR after consolidation, and HD Cytarabine randomization.

**Table 4:** Associations between patients and treatment characteristics and seizures

Covariate	N with seizures/N all patients	% at 20 years (95% CI)	Hazard ratio (95% CI)	p-value
**Protocol**				< 0.001
58741	23/50	45.0 (32.9–61.6)	1	
58831/2	27/189	13.3 (9.3–19.2)	0.29 (0.16–0.51)	
58881	45/596	7.8 (5.9–10.3)	0.15 (0.09–0.25)	
**Age at diagnosis, years**				0.099*
< 6	53/549	9.6 (7.4–12.5)	1	
6–9	29/168	17.5 (12.3–24.8)	1.67 (1.04–2.66)*	
10–17	13/118	10.3 (6.0–17.5)	1.10 (0.60–2.04)*	
**CNS involvement at diagnosis**				0.079*
No CNS involvement	86/785	10.8 (8.8–13.3)	1	
CNS involvement	8/44	18.3 (9.8–34.2)	1.95 (0.93–4.09)*	
VHR after consolidation**				0.143
Not VHR	37/535	7.2 (5.3–9.8)	1	
VHR	6/46	13.2 (6.3–27.9)	1.90 (0.80–4.47)	
Randomized HD Cytarabine** ***				
No HD Cytarabine	5/97	5.3 (2.2–12.4)		
HD Cytarabine	7/94	7.7 (3.8–15.7)		

CNS, central nervous system; HD Cytarabine, high-dose cytarabine; VHR, very high risk

*Adjusted for protocol

**Based on the 58881 study only

***A formal comparison using the Fine and Gray model was not performed due to the small number of events

Additionally, we observed that seizures still occur beyond 5 years of follow-up. The cumulative incidence continues to increase in particular in patients from the 58741 study but also in patients from the 58831/2 study, with some events observed even beyond 20 years (Fig. 1A).

### Stroke

The cumulative incidence of stroke at 20 years from end of treatment was 16% (95% CI 8–35%) in the 58741 study, 2% (95% CI 1–5%) in the 58831/2 studies and 2% (95% CI 1–4%) in the 58881 study (Fig. 1B). Table 5 presents the incidence of stroke by age at diagnosis, CNS involvement at diagnosis, VHR after consolidation, and HD Cytarabine randomization. Patients who were 10–17 years of age at diagnosis had a higher incidence of stroke as compared to those < 6 years of age (HR = 3.35, 95% CI 1.31–8.55). The difference between those 6–9 and those < 6 years of age was small and not statistically significant. Stroke continued to occur beyond 5 years of follow-up, in particular in patients from the 58741 study (Fig. 1B).

**Table 5:** Associations between patients and treatment characteristics and stroke

Covariate	N with stroke/N all patients	% at 20 years (95% CI)	Hazard ratio (95% CI)	p-value
**Protocol**				0.002
58741	7/36	16.3 (7.5–35.4)	1	
58831/2	4/172	1.8 (0.6–5.4)	0.16 (0.04–0.59)	
58881	12/583	2.2 (1.2–3.8)	0.15 (0.05–0.45)	
**Age at diagnosis, years**				0.033*
< 6	11/525	1.5 (0.7–3.1)	1	
6–9	4/150	2.9 (1.1–7.7)	1.15 (0.37–3.62)*	
10–17	8/116	7.5 (3.8–14.8)	3.35 (1.31–8.55)*	
CNS involvement at diagnosis**				
No CNS involvement	20/743	2.5 (1.5–3.9)		
CNS involvement	2/41	5.4 (1.4–20.7)		
VHR after consolidation** ***				
Not VHR	9/524	1.8 (1.0–3.5)		
VHR	3/45	6.7 (2.2–19.9)		
Randomized HD Cytarabine** ***				
No HD Cytarabine	1/95	1.2 (0.2–8.4)		
HD Cytarabine	1/91	1.1 (0.2–7.7)		

CNS, central nervous system; HD Cytarabine, high-dose cytarabine; VHR, very high risk

*Adjusted for protocol

**A formal comparison using the Fine and Gray model was not performed due to the small number of events

***Based on the 58881 study only

### Leukoencephalopathy

The cumulative incidence of leukoencephalopathy at 20 years from end of treatment was 62% (95% CI 43–91%) in the 58741 study, 5% (95% CI 2–10%) in the 58831/2 studies and 3% (95% CI 2–5%) in the 58881 study (Fig. 1C). Table 6 presents the incidence of leukoencephalopathy by age at diagnosis, CNS involvement at diagnosis, VHR after consolidation, and HD Cytarabine randomization. Patients who were 10–17 years of age at diagnosis had a higher incidence of leukoencephalopathy as compared to those < 6 years of age (HR = 3.09, 95% CI 1.54–6.22, Table 6). The difference between those 6–9 and those < 6 years of age was small and not statistically significant. Additionally, leukoencephalopathy continued to occur beyond 5 years of follow-up particularly in patients from the 58741 study but also in those from the other trials, with some events observed beyond 20 years from end of treatment (Fig. 1).

**Table 6:** Associations between patients and treatment characteristics and leukoencephalopathy

Covariate	N with leukoencephalopathy /N all patients	% at 20 years (95% CI)	Hazard ratio (95% CI)	p-value
**Protocol**				< 0.001
58741	12/18	62.2 (42.7–90.7)	1	
58831/2	8/129	4.7 (2.1–10.2)	0.07 (0.03–0.18)	
58881	16/479	3.4 (2.1–5.4)	0.04 (0.02–0.09)	
**Age at diagnosis, years**				0.004*
< 6	20/412	4.2 (2.7–6.8)	1	
6–9	6/126	4.8 (2.2–10.4)	0.83 (0.30–2.31)*	
10–17	10/88	10.2 (5.5–19.0)	3.09 (1.54–6.22)*	
CNS involvement at diagnosis**				
No CNS involvement	30/582	4.7 (3.2–6.8)		
CNS involvement	4/38	10.5 (4.2–26.6)		
VHR after consolidation** ***				
Not VHR	13/435	3.0 (1.8–5.1)		
VHR	2/34	5.9 (1.5–22.6)		
Randomized HD Cytarabine** ***				
No HD Cytarabine	3/76	4.0 (1.3–12.2)		
HD Cytarabine	0/71	0 (not estimable)		

CNS, central nervous system; HD Cytarabine, high-dose cytarabine; VHR, very high risk.

*Adjusted for protocol

**A formal comparison using the Fine and Gray model was not performed due to the small number of events

***Based on the 58881 study only

## Discussion

In this retrospective evaluation, we reported the neurological outcome of 890 childhood ALL survivors treated in three consecutive EORTC CLG trials between 1971 and 1998, with a median follow-up of 19 years.

The starting point of this evaluation was the clinical point of view, and we were particularly interested in late neurotoxicity, which occurs after treatment has been discontinued and/or persists as a result of more structural, long-term neurological damage.

Follow-up of patient will occur at least for 5 years after remission, until the child is cured. Side effect may not be detectable for decade and thus long-term follow-up should be offer to every patient. Indeed, when a child is treated, in particular at a young age, his neurodevelopment is not finished yet so some learning difficulties will not even be detectable. Additionally, some therapy in particular radiation can cause side effect in the long term, i.e., delayed cognitive impairment and stroke. The precise mechanisms responsible for radiation neurotoxicity are not fully understood. Several mechanisms have been suggested such as DNA damage through the production of highly reactive free radical that led to cell death. Other mechanisms for toxicity include reduction of neurogenesis or death of endothelial cell causing thrombus formation on the exposed matrix and vessel obstruction. Finally accelerated atherosclerosis and microangiopathy can lead to vascular insufficiency and infarction. Complications after radiotherapy can be delayed by many years or even decades. In this work, we highlight that a neurological event can occur several years after the treatment, which supports the need of a long-term follow-up and screening [21].

### Cognitive disturbance

Neurocognitive disturbance rates reported here are in concordance with previous work published by St. Jude’s team [3]. Impairment rates across neurocognitive domains at mean 26 years since diagnosis ranged from 28.6 to 58.9% in a large cohort of SCALL [3]. Neurocognitive disturbance may occur in several domains such as executive function, attention, memory and processing speed [3, 4].

We found that neurotoxic complications are far more prevalent in the 58741 cohort, and this could be explained by the CRT applied to treat every patient.

In others previous studies, the predominant role of CRT in long-term neurotoxicity was already demonstrated [4, 5]. Perhaps, CRT may be a key risk factor of neurotoxicity, but the high cumulative dose of steroid therapy (Table S2) and higher single dose of HD MTX may have also played a role. Unfortunately, the design of this study and the number of events do not allow to confirm the impact of each therapy as risk factor.

We also previously reported the long-term outcomes of the randomization comparing *no CRT* versus *CRT* in medium/high-risk patients included in study 58832, at a median follow-up of 20 years (range 4–32 years) [22]. Omission of CRT was associated with significant decrease in the rate of CNS toxicities: leukoencephalopathy and cognitive disturbance were halved, seizures were reduced fourfold, and no patients randomized to the *no CRT* arm had strokes compared with almost 8% of the patients in the *CRT* arm.

Neurotoxicity is widely attributed to CRT [3] but the intensified intravenous administration of chemotherapeutics drugs and ITTs have gradually replaced CRT for the treatment of ALL in children. It was shown that even patients treated with chemotherapy only can also present neurocognitive disturbance mainly in the executive and attention domains [3, 4]. 

### Seizures and stroke

The reported prevalence of seizure/stroke in irradiated patients (25 Gy) from the cohort 58741 is particularly high (45%/16%, respectively) but this study is, to our knowledge, the first to report incidence of seizure/stroke in a SCALL cohort who have all been irradiated. Rate of seizure/stroke in 58831/2 and 58881 cohorts is concordant with previous publications [9, 10, 25]. The 10-year cumulative incidence of subsequent seizure and/or stroke in SCALL was estimate at 4.3% [9]. Neuropsychological performance in patients who experience seizure indicated problems in attention; working memory and processing speed and treatment related seizure were associated with leukoencephalopathy [25]. Seizures can be caused by MTX (such as transient stroke like syndrome) but also due to asparaginase treatment [10]. The long term neurological consequence of this acute toxicity remains to be clarified and is difficult to evaluate separately from other neurotoxic drugs.

### Leukoencephalopathy

We reported here a high rate of leukoencephalopathy in the cohort 58741 and to our knowledge, there is no other study including only irradiated SCALL patients. On the contrary, quite low rates of leukoencephalopathy are reported here, in cohort 58831/2 and 58881 in comparison with previous work [12]. In this study, the authors reported leukoencephalopathy in 23.6% of survivors of SCALL (87 out of 369) among which 14 were symptomatic. Leukoencephalopathy persisted at end of therapy in 74% and 58% of asymptomatic and symptomatic patients respectively. Leukoencephalopathy was prospectively assessed by MRI at four timepoints through treatment.

The difference may be explained by the fact that here the event of leukoencephalopathy was retrieved from medical records and has not been prospectively assessed by magnetic resonance imaging. We may have missed asymptomatic leukoencephalopathy, including in the non-irradiated patient.

Patients above 10 years old at the time of treatment seemed to be more at risk of leukoencephalopathy. This is also consistent with previous work [12]. Methotrexate can also cause long-term leukoencephalopathy. Leukoencephalopathy secondary to methotrexate persisted in 74% of asymptomatic and 58% of symptomatic patients at end of therapy [12] and was associated with decreasing neurocognitive performance [24]. Higher cumulative number of ITTs or high dose IV cytarabine are other risk factors for leukoencephalopathy. [12, 13]

In our previous work, we also showed that decrease in MTX dosage was associated with lower rates of leukoencephalopathy and cognitive disturbance, highlighting the role of chemotherapy in CNS toxicities [24]. 

Finally, if de-escalation in ALL treatment in particular the abandonment of CRT therapy has led to less neurological complications, survivors who received chemotherapy still suffered from apparent cognitive impairment in particular in their attention and executive function domains [4]. In the future, late neurological consequences of new therapy in acute lymphoblastic leukemia such as CAR-T cell therapy or blinatumomab should be carefully monitored. Additionally, the use of dexamethasone in current trial (e.g. https://clinicaltrials.gov/study/NCT04307576) may provide evidence about its additional neurotoxicity.

### Limitations

Due to insufficient data related to second neoplasms (SN) in the 890 patients included, we could not include an analysis of secondary CNS tumours. We previously reported the incidence of SN in 58832 and 58881 EORTC CLG studies and found low incidence of SN and secondary CNS tumours, in particular in patients treated without CRT [22, 25]

Other limitations include the retrospective design of the study, based on medical records and without systematically reassessment of the patients for the purpose of this study. Some late neurological events may therefore have been underreported. Among 2329 eligible patients, only 890 patients were included in the analysis and information about some endpoints was missing among patients included in the analysis. Although patients with and without available data were similar in terms of the risk group and exposure to most toxic treatments, such a large extent of missing data involves a significant risk of a selection bias. Finally, due to the study design, we were not able to investigate the effects of particular treatments.

### Conclusion and perspective

This retrospective observational study highlights the frequency of neurological late effects in SCALL. With the increase of the overall survival of ALL patients in the past decades, late effect surveillance and their management became an important public health challenge. With this regard, our observation that neurotoxicity continues to occur beyond 5 years of follow-up is of particular importance for clinicians involved in late effects clinics. This is a further argument in favour of comprehensive, multidisciplinary long-term follow-up, not limited to the period when initial cancer is treated and most relapses occur.

The role and potential benefit of longitudinal neurological screening such as MRI and/or neuropsychological tests should be evaluated in SCALL, as neurological late effects continue to occur beyond 5 years after end of treatment.

### Supplementary Information

Additional file 1: Figure S1. Flow chart of patients. Table S1. Main characteristics of the first-line treatments according to the EORTC protocols. Table S2. Patients and treatment characteristics by the availability of long-term neurotoxicity data.

## Data Availability

According to the EORTC Data Sharing policies, data are available under specific conditions (please refer to https://www.eortc.org/data-sharing/).
