# Supplementary material for: Long-term neurotoxicity among childhood acute lymphoblastic leukaemia survivors enrolled between 1971 and 1998 in EORTC Children Leukemia Group studies
Source: Discov Oncol. 2024 Jan 29;15:20. doi: 10.1007/s12672-024-00869-6 (PMC10825101; doi:10.1007/s12672-024-00869-6)
Supplement: Supplementary file 1 — Additional file 1: Figure S1. Flow chart of patients. Table S1. Main characteristics of the first-line treatments according to the EORTC protocols. Table S2. Patients and treatment characteristics by the availability of long-term neurotoxicity data. [file 12672_2024_869_MOESM1_ESM.docx]

# Supplementary material





Figure S1. Flow chart of patients.

Table S1. Main characteristics of the first-line treatments according to the EORTC protocols.

| **EORTC study** | **58741** | **58831**^a^ | **58832**^b^ | **58881 LR-IR**^c^ | **58881 VHR**^d^ |
| --- | --- | --- | --- | --- | --- |
| **Treatment start period** | 1971-1978 | 1983-1989 | 1983-1989 | 1989-1998 | 1989-1998 |
| **CRT (dose)** | Yes (25 Gy) | No | No vs yes^e^ | No | No |
| **HSCT** | No | No | No | No | In patients in first remission, if a donor was available |
| **Prednisone (mg/m²)** | 8,120 to 13,800^f^ | 1,680 | 1,680 | 1,680 | 1,680 |
| **Dexamethasone (mg/m²)** | 0 | 140 | 280 | 210 | 700 |
| **MTX ITT (number)** | 5^g^ | 6^g^ | 7^g^ | 10^g^ | 9^g^ |
| **Triple ITT (number)** | 0 | 0 | 0 | 0 | 7^g^ |
| **Asparaginase (U/m²)** | 1,200,000 | 14,5000 | 14,5000 | 120,000 | 420,000 |
| **MTX (mg/m²)** | 480 to 3,915 ^f,h^ | 3,560^i^ | 11,560^i^ | 21,560^i^ | 50,000 |
| **Cytarabine (mg/m²)** | 0 | 1,800 | 1,800 | 1,800 to 9,800^j^ | 44,000 |

^a^standard-risk patients; ^b^medium and high-risk patients; ^c^low-risk and intermediate-risk patients; ^d^very-high-risk patients; ^e^randomized question, dose according to age: 16 Gy (< 1 year), 20 Gy (1-< 2 years), 24 Gy (≥ 2 years); ^f^depending on randomizations (consolidation B versus consolidation A and maintenance immunotherapy versus maintenance chemotherapy); ^g^dose according to age (see Supplementary Figure S1); ^h^calculated based on a 3-years maintenance duration at the theoretical dose of 15 mg/m²/week; ^i^calculated based on a 1.5-years maintenance duration at the theoretical dose of 20 mg/m²/week; ^j^according to randomization (high-dose Cytarabine versus no high-dose Cytarabine). Abbreviations: CRT, cranial radiotherapy; Cytarabine, cytarabine; HSCT, hematopoietic stem cell transplantation; IR, intermediate-risk; ITT, intrathecal therapy; LR, low-risk; MTX, methotrexate; VHR, very high risk.

Table S2. Patients and treatment characteristics by the availability of long-term neurotoxicity data.

|  | **Data missing for all outcomes (N=1439)** | **Data included in the analysis (N=890)** |
| --- | --- | --- |
|  | **N (%)** | **N (%)** |
| **Protocol** |  |  |
| **58741** | 99 (6.9) | 60 (6.7) |
| **58831/2** | 442 (30.7) | 199 (22.4) |
| **58881** | 898 (62.4) | 631 (70.9) |
| **Sex** |  |  |
| **Male** | 818 (56.8) | 471 (52.9) |
| **Female** | 621 (43.2) | 419 (47.1) |
| **Age at diagnosis, years** |  |  |
| **<6** | 848 (58.9) | 580 (65.2) |
| **6-9** | 297 (20.6) | 181 (20.3) |
| **10-17** | 294 (20.4) | 129 (14.5) |
| **WBC at diagnosis, x 10⁹/l** |  |  |
| **Patients with available data** | 1436 (99.8) | 890 (100.0) |
| **<25** | 922 (64.2) | 612 (68.8) |
| **25 - <50** | 196 (13.6) | 116 (13.0) |
| **≥50** | 318 (22.1) | 162 (18.2) |
| **CNS involvement at diagnosis** |  |  |
| **Patients with available data** | 1402 (97.4) | 882 (99.1) |
| **No CNS involvement** | 1318 (94.0) | 835 (94.7) |
| **CNS involvement** | 84 (6.0) | 47 (5.3) |
| **NCI risk group** |  |  |
| **Patients with available data** | 1436 (99.8) | 890 (100.0) |
| **Standard Risk** | 890 (62.0) | 618 (69.4) |
| **High Risk** | 546 (38.0) | 272 (30.6) |
| **CRT** |  |  |
| **No** | 1261 (87.6) | 744 (83.6) |
| **Yes** | 178 (12.4) | 146 (16.4) |
| **HSCT** |  |  |
| **No** | 1211 (84.2) | 791 (88.9) |
| **Yes** | 228 (15.8) | 99 (11.1) |
| **Relapse** |  |  |
| **No** | 945 (65.7) | 716 (80.4) |
| **Yes** | 494 (34.3) | 174 (19.6) |
| **CNS relapse** |  |  |
| **No** | 1250 (86.9) | 805 (90.4) |
| **Yes** | 189 (13.1) | 85 (9.6) |
| **Survival status** |  |  |
| **Alive** | 1047 (72.8) | 804 (90.3) |
| **Dead** | 392 (27.2) | 86 (9.7) |

CNS, central nervous system; CRT, cranial radiotherapy; HSCT, hematopoietic stem cell transplantation; NCI, National Cancer Institute; WBC, white blood cells.
